# A trypsin-like protease with apparent dual function in early *Lepeophtheirus salmonis *(Krøyer) development

**DOI:** 10.1186/1471-2199-10-44

**Published:** 2009-05-13

**Authors:** Rasmus Skern-Mauritzen, Petter Frost, Sussie Dalvin, Bjørn Olav Kvamme, Ingunn Sommerset, Frank Nilsen

**Affiliations:** 1 Department of Population Genetics and Ecology, Institute of Marine Research, 5817 Bergen, Norway; 2 Intervet Norbio AS, 5008 Bergen, Norway; 3 Department of Health, Institute of Marine Research, 5817 Bergen, Norway; 4 Department of Biology, University of Bergen, 5020 Bergen, Norway

## Abstract

**Background:**

Trypsin-like serine proteases are involved in a large number of processes including digestive degradation, regulation of developmental processes, yolk degradation and yolk degradome activation. Trypsin like peptidases considered to be involved in digestion have been characterized in *Lepeophtheirus salmonis*. During these studies a trypsin-like peptidase which differed in a number of traits were identified.

**Results:**

An intronless trypsin-like serine peptidase (*LsTryp10*) from *L., salmonis *was identified and characterized. *LsTryp10 *mRNA is evenly distributed in the ovaries and oocytes, but is located along the ova periphery. LsTryp10 protein is deposited in the oocytes and all embryonic cells. *LsTryp10 *mRNA translation and concurrent degradation after fertilization was found in the embryos demonstrating that LsTryp10 protein is produced both by the embryo and maternally. The results furthermore indicate that LsTryp10 protein of maternal origin has a distribution pattern different to that of embryonic origin.

**Conclusion:**

Based on present data and previous studies of peptidases in oocytes and embryos, we hypothesize that maternally deposited LsTryp10 protein is involved in regulation of the yolk degradome. The function of LsTryp10 produced by the embryonic cells remains unknown. To our knowledge a similar expression pattern has not previously been reported for any protease.

## Background

Trypsin-like serine peptidases of the S1A subfamily (hereafter referred to as S1A peptidases) are found in all metazoan groups and are involved in a variety of biological processes [1,2]. They are synthesized as inactive zymogens which are activated by proteolytic cleavage at a defined site N-terminal to the proteolytic domain. They may consist of the proteolytic domain only (referred to as single domain peptidases) or may contain one or more additional domains, generally N-terminal to the proteolytic domain (referred to as multi domain peptidases). S1A peptidases are generally extracellular peptidases although some have intracellular functions [2,3]. S1A peptidases involved in digestion commonly consist of a proteolytic domain only, but single domain S1A peptidases may exhibit strict specificity and serve regulatory roles [4-6]. However, regulatory S1A peptidases generally include one or more additional domains [2,5,7]. Trypsins are S1A peptidases with a specific architecture that cleave substrates after Arg and Lys [2]. They are common digestive enzymes in metazoans and their zymogens are activated by trypsins or enteropeptidases [2]. Once activated, the digestive trypsins contribute to the proteolysis of ingested proteins and also activate other digestive zymogens such as chymotrypsinogens and proelastases [2].

S1A peptidases also play important roles during fertilization and early development; at fertilization S1A peptidases are necessary to prevent polyspermy by catalyzing formation of a fertilization envelope [8,9] and in the early embryo, S1A peptidases participate in developmental control [10,11] and cell migration [12,13]. Egg yolk degradation has been reported to be catalyzed both by aspartic and cysteine cathepsins and serine peptidases, including S1A peptidases [14-20]. S1A peptidases involved in egg yolk degradationare suggested mainly to serve in degradome activation [14,17,21], but it should be noted that the main mechanism controlling activation of yolk degrading proteases appears to be decreasing yolk granule pH [14,15,22].

Proteins needed before the midblastula transition are supplied maternally or encoded by maternal mRNA. S1A peptidases exerting their role in the embryos prior to the midblastula transition appear to be transcribed and translated either maternally [23] or by germ line cells other than the oocyte [10]. Proteases involved in, or putatively involved in, yolk degradation generally appear to be translated before fertilization [24-26] although embryonic transcription and translation of a vitellogenin degrading S1A peptidase has been reported in *Bombyx mori *[18]. However, the exact spatiotemporal patterns of transcription and translation are unknown for many peptidases active during early development. Maternal transcripts have been reported to comprise transcripts from more than 50% of the protein encoding genes in an organism [27,28]. However, detailed studies of maternally encoded S1A peptidases have not been reported previously.

Salmon lice (*L. salmonis*) are obligate ectoparasites on salmonid fishes and a major pest in salmon aquaculture. Adult female salmon lice are fertilized by males that deposit spematophores at the genital segment [29]. At regular temperature dependent intervals (approximately 10 days at 10°C (personal observations)) they produce a pair of eggstrings (containing ≤ 1500 eggs) that are fertilized when they are extruded [29]. During characterization of trypsins and trypsin-like peptidases in *L. salmonis *[30-33] we identified an intronless single domain trypsin that was transcribed by adult female *L. salmonis *(*LsTryp10*, accession# EF490878). In the present study *LsTryp10 *is characterized and functional implications are discussed.

## Results

### Sequence analysis

EST sequencing led to identification of a 1052 b.p. transcript encoding a serine proteinase named LsTryp10. The transcript consist of a 810 b.p. ORF, a 37 b.p. 5' untranslated region (UTR) and a 205 b.p. 3' UTR. Alignment of genomic and corresponding cDNA sequences revealed that *LsTryp10 *is devoid of introns. The protein was predicted to be secreted from the cell using the analytical strategy recommended by Emanuelsson et al. [34]. The encoded 270 amino acid (a.a.) protein with a predicted weight of 30 kDa consist of a putative 17 a.a. signal peptide, a 10 a.a. activation peptide and a 243 a.a. proteolytic domain. The proteolytic domain in the predicted protein includes the catalytic triad His57, Asp102 and Ser195 in a sequence context typical for proteinases of the S1A subfamily in addition to residues characteristic for S1A peptidases with trypsin specificity, including Tyr172, Asp189, Gln192, Gly216 and Gly226 (chymotrypsinogen numbering [35]). The encoded trypsinogen appear to contain the 3 cysteine bridges conserved in S1A peptidases [36] and an additional cysteine bridge between Cys106 and Cys129. However, LsTryp10 do not contain the disulphide bridge (Cys59 to Cys104) conserved in previously described single domain trypsins from *L. salmonis *[31]. The overall identity to previously published trypsins from *L. salmonis *[30,31,37] was ≤ 33%.

### Phylogenetic analysis

**Figure 1:**
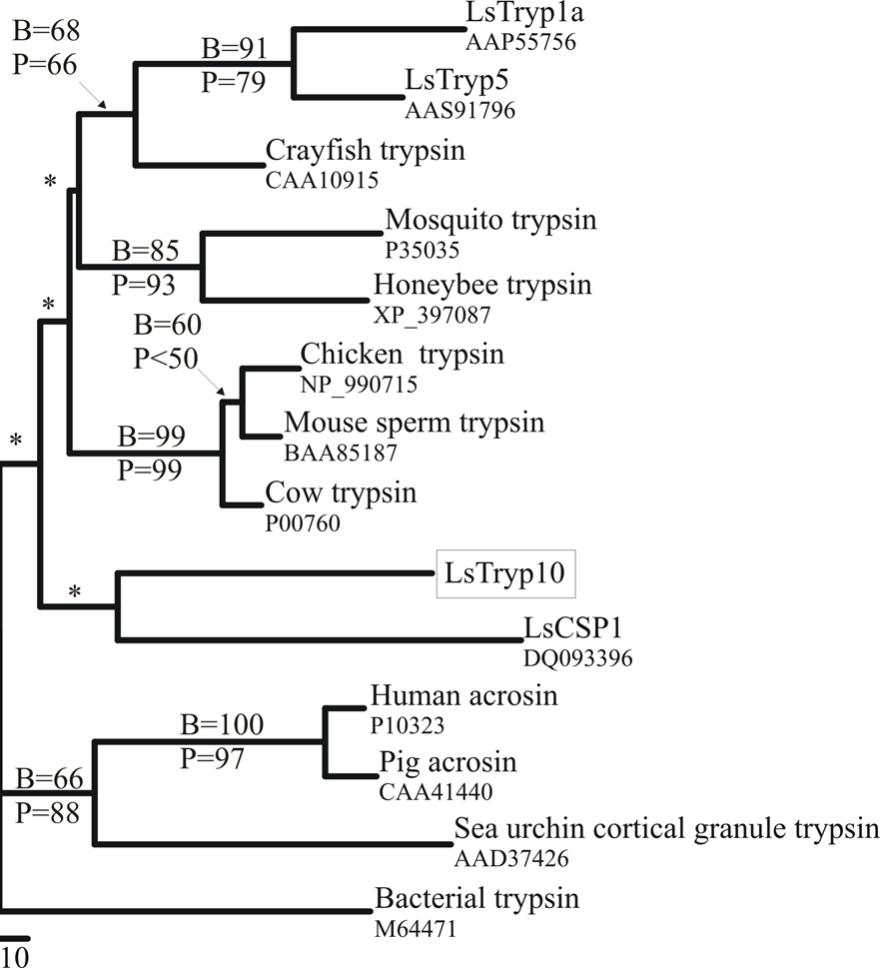
**Maximum likelihood tree**. The tree was generated by the Proml module in the Phylip package. Phylogenetic analysis of selected S1A peptidases. Support was calculated by bootstrapping (B) and quartet puzzling (P). Unsupported branches are indicated by *. Bacterial trypsin was used as outgroup. Accession numbers are indicated in the tree.

The phylogenetic analysis shows that vertebrate, crustacean and insect trypsins reside on separate well-supported branches (Fig. 1). Furthermore the vertebrate acrosins, found in sperm cells, form a distinct group within a well-supported monophyletic clade that includes Cortex Granule Serine Protease 1 (*CGSP1*) found in sea urchin eggs. Most of the internal nodes in the phylogenetic tree were not supported (Fig. 1) due to the high frequencies of highly conserved and highly divergent residues resulting in relatively low prevalence of informative semi-conserved residues. *LsTryp10 *does not appear to be closer related to previously characterized *L. salmonis *single domain trypsins [31] than to any of the other supported trypsin branches. It should be noted that although *LsTryp10 *resides on the same branch as the clip domain containing serine peptidase *LsCSP1 *[33] this position is not supported (Fig. 1).

### Stage specific LsTryp10 transcription and size confirmation

**Figure 2:**
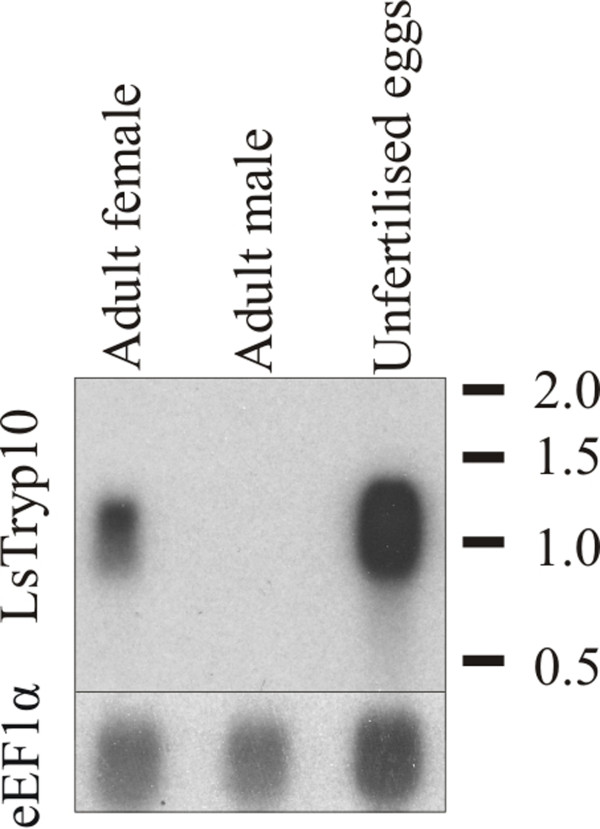
**Northern blot analysis of LsTryp10 and eEF1α**. Northern blot analysis showing the size of LsTryp10 and relative LsTryp10 and eEF1α transcript levels in adult females, males and unfertilized eggs. Loaded total RNA was approximately 4.5 μg RNA pr. well. Exposure times for LsTryp10 and eEF1α are dissimilar.

**Figure 3:**
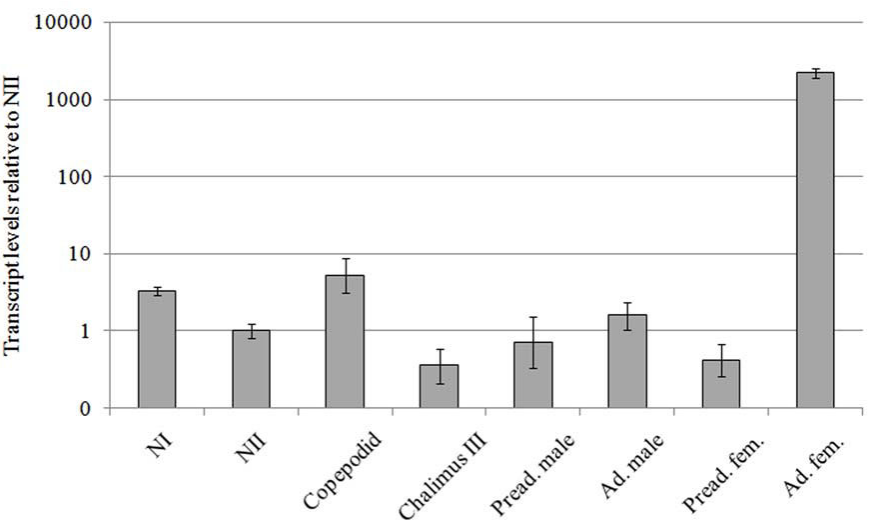
**LsTryp10 transcript levels**. LsTryp10 transcript levels at different developmental stages quantified relative to the level found in the nauplius II (NII) stage. Error bars show 95% c.i. calculated from the ΔΔC_T _values from each dilution. The error bars for NII show the 95% c.i. calculated from the ΔC_T _values. Note the logarithmic vertical axis.

A single transcript of approximately 1200 b.p. was identified in the northern hybridization analysis (Fig. 2). This corresponds well with the 1073 b.p. cDNA sequence. In northern blot analyses *LsTryp10 *mRNA was undetectable in adult males but present in mature females and unfertilized eggstrings (Fig. 2). This pattern was confirmed by Q-PCR analyses where *LsTryp10 *mRNA was present at background levels in preadult stages and adult males, but was significantly upregulated in adult females (Fig. 3). Microarray analysis have furthermore shown that *LsTryp10 *transcript abundance increase during development after the last molt in females (Contig 328 in Eichner et al. [38]).

### Localization of LsTryp10 mRNA

**Figure 4:**
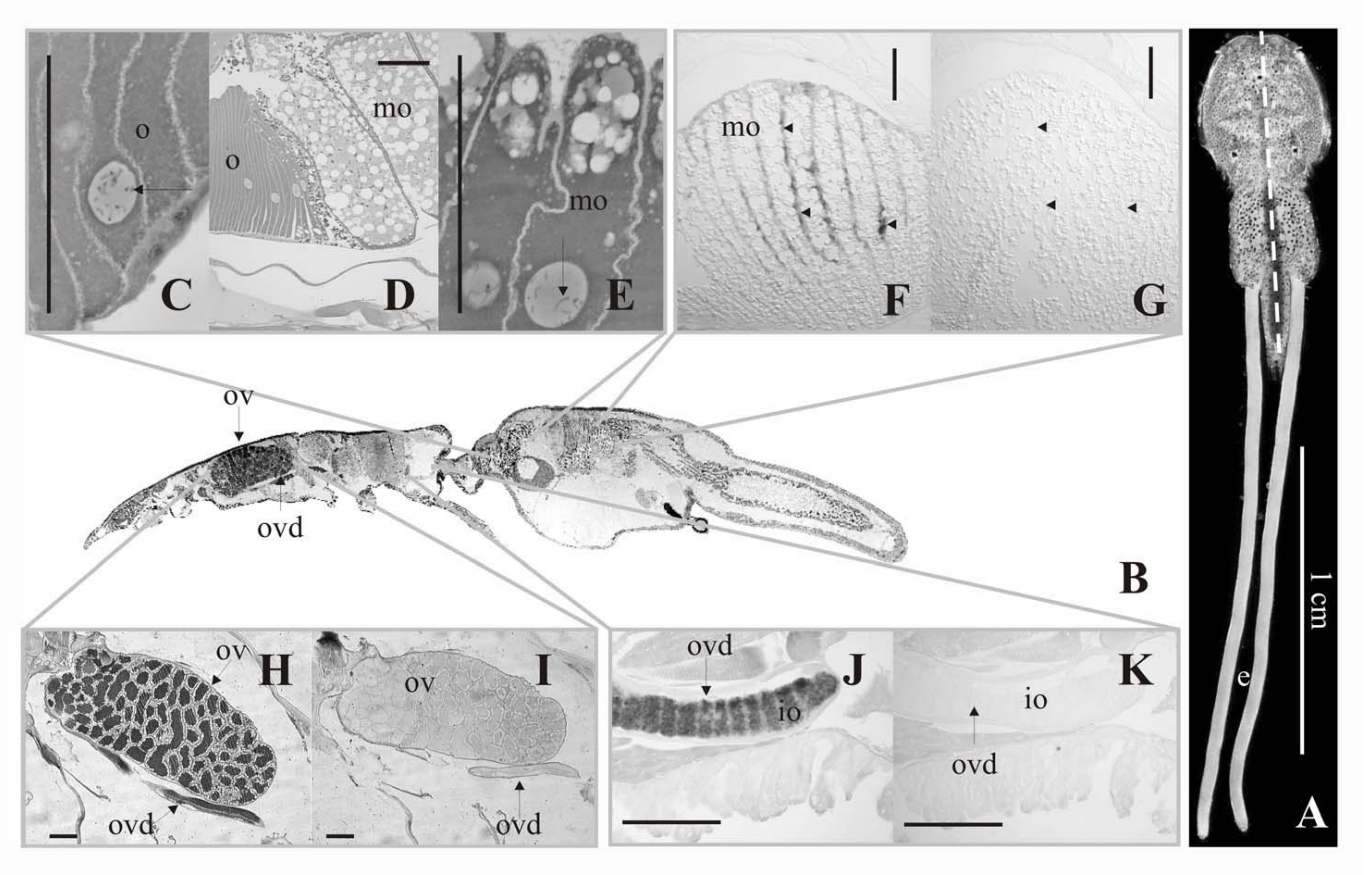
**LsTryp10 mRNA localization and histology**. **A**. Adult female *L. salmonis *with eggstrings, broken white line indicates location of section shown in **B**. e: eggstrings. **B**. Section of adult female *L. salmonis*. ov: ovary; ovd: oviduct. **C**. Immature oocytes (o) in the oviduct. **D**. Transition from immature oocytes in the oviduct (**C**) to mature oocytes in the genital segment (**E**). **E**. Mature oocytes (mo) in the genital segment after being filled with droplets and granules. Note that the chromosomes appear to be in the pachytene or diplotene stage both in the immature and mature oocytes (arrows in **C **and **E**). ISH using antisense (**F, H, J**) and sense (**G, I, K**) RNA probes show that LsTryp10 mRNA is present in the ovary (ov), in immature oocytes (io) in the oviduct (ovd) as well as in mature oocytes in the unfertilized eggstring (ufe). In the mature oocytes LsTryp10 mRNA is localized along the oocyte periphery (arrowheads in **F**, corresponding locations in negative control (**G**) also marked with arrowheads). Bar = 1 cm in **A**, 100 μm in **C**-**K**.

*In situ *hybridization showed that *LsTryp10 *mRNA was present in the ovary and in the oocytes throughout the oviduct (Fig. 4F–J). *LsTryp10 *mRNA is homogenously dispersed in the oocytes localized in the anterior part of the oviducts in cephalothorax. Upon entry into the genital segment the oocytes expand substantially in size, apparently due to incorporation of granules and lipids, and *LsTryp10 *mRNA becomes localized to the periphery of the ova (Fig. 4F).

### Yolk lumen acidification, LsTryp10 protein localization, translation and mRNA degradation

**Figure 5:**
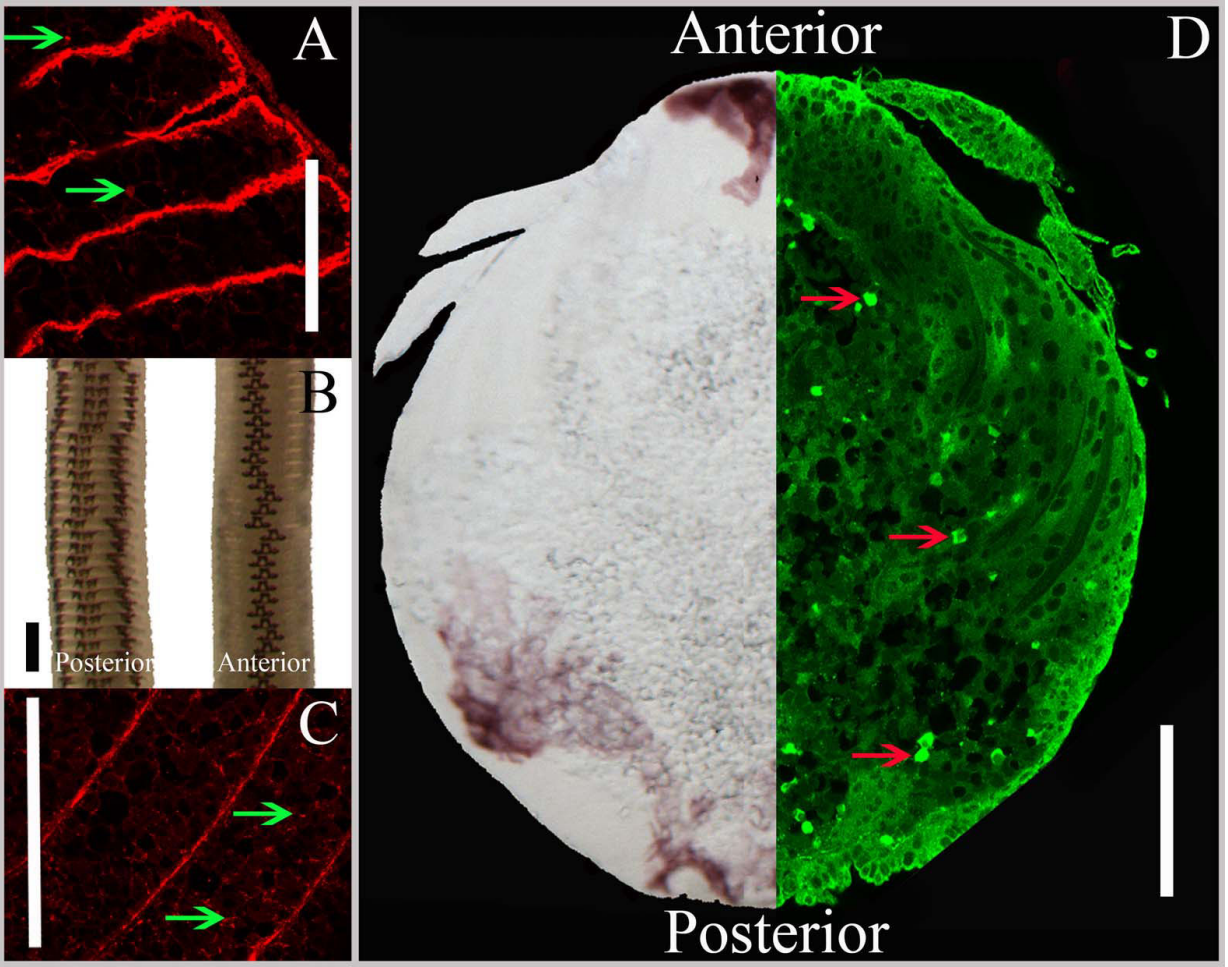
**Immunolocalization of LsTryp10**. **A **and **C**: Confocal section showing the peripheral and yolk associated (green arrows) localization of LsTryp10 (red) in unfertilized oocytes within adult females (**A**) and in fertilized eggs 30 minutes after fertilization (**C**). **B**: Fertilized eggstrings 8 days after fertilization. **D**: Left side: Boiled embryo 5 days after eggstring fertilization. Note the localization of the yolk compartment (granular content). Right side: Confocal section showing localization of LsTryp10 (green) in the embryonic tissue and associated with yolk granules (red arrows). Bars indicate 225 μm in **B **and 100 μm in **A**, **C **and **D**.

**Figure 6:**
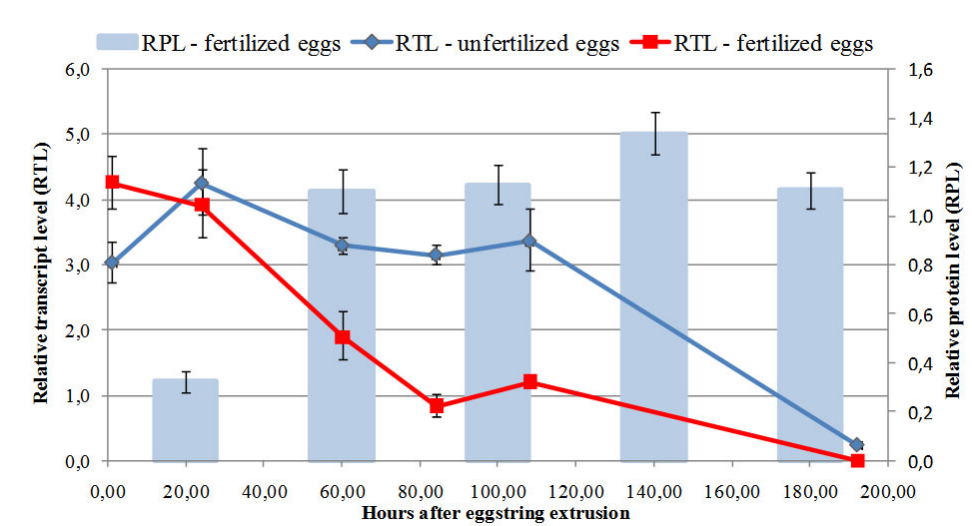
**Relative protein and mRNA levels in developing embryos**. Relative LsTryp10 encoding transcript levels in unfertilized and developing fertilized eggs (RTL – see text for details) determined by Q-PCR. The connective lines are added for clarity and do not necessarily reflect transcript level progression. The relative LsTryp10 protein levels (RPL) in developing fertilized eggs determined by ELISA studies are also shown. Error bars indicate 95% c.i.

LsTryp10 protein was found in embryos and unfertilized mature eggs. In the unfertilized oocytes inside the genital segment LsTryp10 was distributed along the oocyte periphery and in patches associated with yolk granules (Fig. 5A). After fertilization, LsTryp10 was also shown to be present throughout the embryonic cells and in patches associated with yolk granules (Fig. 5C, D). Negative controls were performed without primary antibodies and confirmed specific staining. Relative quantification showed that the LsTryp10 protein level in the fertilized eggs increased between 1 hour and 48 hours after fertilization and then remained stable, whereas the encoding *LsTryp10 *mRNA level decreased steadily from 1 to 192 hours after fertilization (Fig. 6). In contrast the *LsTryp10 *mRNA level in unfertilized eggs appeared relatively stable between 1 and 108 hours after extrusion and then decreased to a level comparable to that found in fertilized eggs 192 hours after fertilization (Fig. 6). The yolk compartment was shown to be acidic at day 5 after fertilization by exposing embryos to LysoTracker, whereas no indications of acidic compartments were identified elsewhere in the embryo (results not shown).

## Discussion

The results show that *LsTryp10 *encodes a peptidase that includes the catalytic triad residues (His, Asp and Ser) in a context typical for serine peptidases of the S1A clan in addition to the specificity determinants for trypsin [4]. Transcript length and presence was confirmed by northern blot analysis (Fig. 2). Interestingly, *LsTryp10 *is devoid of introns, which is a feature also reported for functional digestive trypsins in *Anopheles gambiae *[39]. The phylogenetic analysis do not indicate a reliable phylogenetic position for *LsTryp10*, which appears no closer related to other single domain trypsins from *L. salmonis *[30,31,33,37] than to bacterial trypsin or the clip domain containing *LsCSP1 *[33]. *CGSP1*, which is thought to activate the fertilization envelope and prevent polyspermy in sea urchins [8], appears to be closer related to acrosins than to the other serine peptidases included in the analysis. Since this may give the impression that S1A peptidases related to fertilization form a monophyletic assemblage it should be noted that mouse *TESP4 *which is suggested to have a fertilization related function [40], resides on a well supported branch with pancreatic trypsin from cow and chicken (Fig. 1).

Background *LsTryp10 *transcription is detected in all investigated *L. salmonis *stages except adult females where it is upregulated more than 2000-fold relative to the level in nauplius II larvae (Fig. 3). *In situ *hybridization results show that *LsTryp10 *mRNA is transcribed in the ovary. *LsTryp10 *transcripts are evenly distributed in the immature oocytes in the anterior oviduct in cephalothorax (Fig. 4H, J), but are located along the periphery of the ova in the genital segment (Fig. 4F). The change in distribution of *LsTryp10 *mRNA indicates that the transcripts are actively localized in the egg, as are the transcription factor *bicoid *mRNA in *Drosophila melanogaster *and the transforming growth factor *Vg1 *mRNA in *Xenopus laevis *[41,42]. The 3' UTR in *LsTryp10 *mRNA is substantially longer (205 b.p.) than the 3'-UTRs (44–104 b.p.) in previously characterized *L. salmonis *S1A peptidases [30,31,33,37]. Considering that localization of many transcripts, including *bicoid *mRNA and *Vg1 *mRNA, depends on elements in their 3' UTRs [41-45] the relatively long *LsTryp10 *mRNA 3' UTR may contain elements involved in control of mRNA localization and translation.

The distribution of *LsTryp10 *mRNA suggests that it is a maternal transcript. This notion appears to be supported by the simultaneous *LsTryp10 *mRNA translation and degradation after fertilization (Fig. 6). However, immunohistochemical studies demonstrate this perception to be incorrect as LsTryp10 protein is found in oocytes inside the female's genital segment as well as in most embryonic cells (Fig. 5). The question then arises: Is the embryonic LsTryp10 protein encoded by maternal mRNA? The first cleavage in order Siphonostomatoida (which includes *L. salmonis*) is holoblastic but strongly unequal with the smaller cell giving rise to the embryonic cells and the larger cell containing most of the yolk [46-48]. A similar development in *L. salmonis *must be expected which implies that only a small fraction of the deposited maternal mRNA is present in the embryonic cells. The stable level of *LsTryp10 *mRNA in developing eggs (Fig. 6) may thus be the product of a simultaneous degradation of maternal *LsTryp10 *mRNA in the large yolk containing cell and zygotic transcription of *LsTryp10 *mRNA in the developing embryonic cells [47-49]. The very low expected levels of maternal LsTryp10 encoding transcripts inside the embryonic cells thus strongly indicates that LsTryp10 protein translated after fertilization is encoded by zygotically transcribed *LsTryp10 *mRNA. It is plausible that the maternal and zygotic *LsTryp10 *mRNAs are encoded by paralog genes.

In the oocytes, maternally deposited LsTryp10 protein (mLsTryp10) was found along the periphery and in patches associated with vitellogenin granules inside the yolk compartment (Fig 5). This yolk associated localization resembles the reported distribution of an *Artemia *trypsin-like peptidase with a putative degradome activating function [50] as well as other peptidases putatively involved in yolk degradation or degradome activation [17,51,52]. Earlier reports suggest, that serine peptidases are not directly responsible for yolk degradation but activate a second set of peptidases (generally cathepsins) that perform the degradation of the yolk [14,17,21]. A similar function for mLsTryp10 protein is likely. Yolk degradation appear to be initiated by yolk granule acidification in many invertebrate species [15,22,52,53]. This may also be the case in *L. salmonis *as we observed acidic conditions in the yolk compartment in 5 days old embryos. It is possible that acidification leads to autoactivation of mLsTryp10 protein that in turn activates the general yolk degradome. The LsTryp10 protein translated by the embryo (eLsTryp10) is found in all embryonic cells, including cells in the antennae (Fig. 5D) which indicate that eLsTryp10 has a function not related to egg yolk degradation. Based on the ubiquitous distribution of eLsTryp10 it is likely to be involved in general developmental processes such as modification of the cell-matrix e.g. associated with cell movement [12,13]

In conclusion, LsTryp10 is a trypsin which is both deposited in the oocytes and translated after fertilization. Maternally deposited and embryonic LsTryp10 seems to be translated from different pools of mRNA; one maternally transcribed and one transcribed after fertilization. The deposited LsTryp10 is likely to be involved in egg yolk degradation whereas embryonic LsTryp10 appears to serve a different purpose, e.g. cell matrix modification. LsTryp10 is the first arthropod peptidase reported to be both maternally deposited and translated in developing embryos.

## Conclusion

An intronless trypsin-like serine peptidase, LsTryp10, was characterized in *L. salmonis*. *LsTryp10 *mRNA was significantly upregulated in adult females relative to all other investigated stages. *LsTryp10 *mRNA was found in the ovaries, oviducts, ova and developing embryos. The encoded protein was found in ova as well as in developing embryos where it was also shown to be translated. Considering the pattern of the first cell divisions in *L. salmonis*, the present results indicate that LsTryp10 proteins in the embryos stem from two distinct lines of synthesis. First maternally transcribed and translated LsTryp10 protein is deposited in the oocytes and second embryonically transcribed LsTryp10 mRNA is translated in all embryonic cells. The maternally produced LsTryp10 protein is likely to be involved in yolk degradation or yolk degradation control, whereas the function of embryonically produced LsTryp10 protein is unknown.

## Methods

### Handling and sampling of fishes and parasites

*L. salmonis *were cultured on Atlantic salmon (*Salmo salar *(Linnaeus)) as described previously [31]. *L. salmonis *for quantitative real-time PCR (Q-PCR) analysis were sampled as nauplius I larvae (n≈10000), nauplius II larvae (n≈10000), infectious copepodids (n≈5000), chalimus III (n = 35), preadult I&II males mixed (n = 10), preadult I females (n = 10), adult males (n = 16) and adult females (n = 10). *L. salmonis *for Q-PCR and cDNA sequencing were stored in RNA *later *(Ambion, Huntingdon, UK), for 24 hours at 4°C and then at minus 20°C. Adult females used for isolation of genomic DNA were stored in 70% ethanol. Eggstrings for transcript quantification were sampled from fertilized females and unfertilized females. These were obtained by incubating preadult II females on salmon (not previously infected) with and without adult *L. salmonis *males. The copulative statuses for the groups were confirmed by visual examination for spermatophores. The eggstrings were sampled at 1 hour (± 30 minutes), 24 hours (± 4 hours), 60 and 108 hours (both ± 8 hours) after fertilization and stored at -80°C. Eggstrings for protein analysis were sampled after 1 hour (± 30 minutes) and 48, 72, and 96 hours (all ± 8 hours).

### Sequencing of gDNA and full-length cDNA

Expressed Sequence Tags (ESTs) from *L. salmonis *were obtained as described previously [31] and are described in Eichner et al. [38]. The trypsin encoding EST clone with the longest insert was selected and completely sequenced on both strands using primer walking. Additional 5' sequence was obtained as described previously [31]. Based on the obtained cDNA sequences, primer pairs for genomic PCRs were constructed and the resulting PCR products were cloned and more than 30 clones were completely sequenced on both strands using primer walking. Additional genomic sequence was obtained using the GenomeWalker kit in accordance with the manufacturers instructions (Clonetech, Mountain view, California, USA).

### Northern blot hybridization analyses

Total RNA was purified from unfertilized eggstrings, an adult male and an adult female *L. salmonis *using TRIZOL^® ^following the manufacturers recommendations (Life Technologies, Gaithersburg, Maryland, USA). Northern blotting was performed using approximately 4.5 μg RNA per well and sequence specific [^32^P] labeled DNA probes against the *LsTryp10 *ORF and the homogeneously transcribed *eEF1α *[54]. Membranes were prehybridized 30–60 min. at 68°C in PerfectHyb solution (Sigma-Aldrich Norway, Oslo, Norway). Denatured probes were added and incubation was continued over night. After washing twice for 10 min. at 68°C in 0.1× SSC/0.1× SDS, signals from the membrane were detected using Kodak BioMax MS X-Ray film (Kodak, Rochester, New York, USA).

### Bioinformatic analysis

Sequence handling and editing was done using Vector NTI 9.1.0 (Invitrogen, Carlsbad, California, and USA.) and GeneDoc . For analysis of a plausible protein location the translated *LsTryp10 *sequence was analyzed by submission to TargetP 1.1 [55], SignalP 3.0 [56], TMHMM 2.0 [57], TMBETA-NET [58] and big-PI [59] according to the suggestion by Emanuelsson et al. [34]. Phylogenetic analyses of selected amino acid sequences corresponding to the mature peptidases were performed using the Phylip package [60] and Tree-Puzzle v. 5.2 [61]. Mutation rate heterogeneities were calculated in Tree-Puzzle using the JTT model and 8 rate heterogeneities. The distribution coefficient α was 0.93. A maximum likelihood tree was generated by Proml in the Phylip package (jumbling 10 times) and support was calculated by bootstrapping (100 replicates in Phylip) and puzzling (10000 times in Tree-Puzzle).

### Relative quantification of LsTryp10 mRNA levels

The transcript levels during the *L. salmonis *life cycle and in eggstrings at different ages after fertilization were determined by Q-PCR using ABI 7900 HT (eggs and embryos) and ABI 7700 (adults and larval stages) PCR Systems (Applied Biosystems, Foster City, California, USA) at standard settings. The real-time primers and probe were: Forward primer: TTG CAA GAC CGG AAC AAG AAC. Reverse primer: CAA ATC TGA GTA CAC CCC AAC CT. Probe: 6FAM-CAA CTA CAA GAG GTG TCC CAT CCG GG-TAMRA. The real time assays were performed as previously described [31] with the modification that additional TurboDNase (Ambion, Huntingdon, UK) treatments were performed on the RNA from nauplius I and II, chalimus III and preadult males and females. The results were analyzed by the ΔC_T _approach [62] as described previously [31]. The 95% c.i. values at each developmental stage presented in Fig. 3 were calculated from the average ΔΔC_T _values for each dilution, except for nauplius II (which was used as calibrator thereby defining the average ΔΔC_T _values as 0 for this stage). For nauplius II the 95% c.i. shown in Fig. 3 was calculated from the average ΔC_T _at each dilution. The results were confirmed by analysis using a kinetic approach [63] as earlier suggested [64].

For determination of relative *LsTryp10 *mRNA levels in eggs, total RNA from four pairs of eggstrings for each sampled time point was isolated in TRI REAGENT (Sigma-Aldrich Norway, Oslo, Norway) using a FastPrep homogenizer and Lysing Matrix D (Q-Biogene, Solon, Ohio, USA). Extracted RNA was treated with TURBO DNAse (Ambion, Huntingdon, UK) and added 0.5 U/μl SUPERase• In™ (Ambion, Huntingdon, UK). Excellent quality and integrity of the RNA was confirmed using a Bioanalyzer according to the manufacturers instructions (Agilent, Santa Clara, California, USA.). Concentrations were adjusted to 100 ng/μl following quantification on a NanoDrop ND 1000 spectrophotometer (Thermo Fisher Scientific, Waltham, Massachusetts, USA) and diluted in two fold series from 100 ng/μl to 6.25 ng/μl. The cDNA syntheses and Q-PCRs were conducted independently at each dilution as previously described [31] with the exception that cDNA was made using MultiScribe reverse transcriptase according to the manufacturers recommendations (Applied Biosystems, Foster City, California, USA). Relative expression levels were expressed as ΔC_T _relative to the mean transcript level in all the sampled eggstrings using the following formula:



Where C_T mean*dilution _is the mean of the C_T _values of all samples at a given dilution and C_T sample*dilution _is the mean C_T _value of the sample parallels at a given dilution. The relative transcript levels (RTL) presented in Fig. 5 are calculated as . The c.i. is calculated from the ΔC_T _values from the five dilutions. The results were confirmed by analysis using a kinetic approach [64].

### Identification of LsTryp10 mRNA localization

The localization of *LsTryp10 *mRNA in adult female *L. salmonis *was determined by *in situ *hybridization as described previously [31] using a cDNA clone covering 669 base pairs (b.p.) of the open reading frame (ORF) as template for RNA probe synthesis. Hybridizations were performed with antisense probes to show localization of transcripts and with sense probes as negative controls. Labeled probes were visualized using anti-DIG alkaline phosphatase FAB fragment (Roche, Basel, Switzerland) and a chromogen substrate containing Levamisol (Sigma-Aldrich Norway, Oslo, Norway), NTB and BCIP (Roche, Basel, Switzerland).

### Histological analysis of L. salmonis

Adult female *L. salmonis *were fixed in 4% paraformaldehyde in PBS, dehydrated in ethanol (70%, 80%, 90% and 95%) and subsequently embedded in Technovit 7100 following the manufacturers recommendations (Heraeus, Hanau, Germany). Sections (2 μm) were produced using a microtome, stained with metachromatic toluidin and examined using a Leica DMRBE light microscope (Leica Microsystems, Wetzlar, Germany).

### Relative quantification of LsTryp10 protein levels in fertilized eggs

The relative amounts of LsTryp10 protein in developing fertilized *L. salmonis *eggs were determined by enzyme linked immunosorbent assay (ELISA) using rabbit peptide antiserum raised against the peptide CEMHPGYSTSKQDN conjugated to keyhole limpet hemocyanin (BioGenes, Mannheim, Germany). The eggs (4 eggstring pairs from each sampled age) were cut in small pieces and homogenized by sonication in 250 μl 50 mM Tris, 1 mM NaCl, 1 mM EDTA, pH 10 while on ice. Sonication was performed with a microtip for a total of 60 seconds in short pulses (≤ 20 seconds) followed by centrifugation at 3000 g, 4°C for 20 minutes. The water soluble protein concentration was normalized to 600 μg/ml using the Bio-Rad protein assay kit II as instructed by the manufacturer (BioRad, Hercules, California, USA.). ELISA was conducted according to Sommerset et al. [65] with the following modifications: After each incubation step, except after addition of conjugate, the ELISA plates were washed 3 times with PBST. Nunc-Maxisorb microplates (Thermo Fisher Scientific, Roskilde, Denmark) were coated ON at 4°C with four dilutions (50, 25, 12 and 6 μg/ml) of the normalized protein solution, blocked at RT for one hour with 5% non fat dry milk powder in PBST, and incubated for 2 hours at RT with antiserum diluted 1:100 in PBST containing 1% non fat dry milk powder (Sigma-Aldrich Norway, Oslo, Norway). Bound antibodies were detected by addition of HRP conjugated goat-anti-rabbit ABs (BioRad, Hercules, California, USA, 1:2000 in PBST, 1% non fat dry milk powder (Nestlé, Sandvika, Norway), 1 hour at RT), followed by 6 washes with PBST and incubation with 100 μl o-Phenylenediamine + H_2_O_2 _according to instructions from the manufacturer. The chromogenic reaction was stopped after 20 minutes by addition of 50 μl 2,5N H_2_SO_4_. Absorbances at 492 nm were measured for the above described antisera (Abs_test_) and their corresponding preimmune sera (Abs_bacground_) from the same plates. The corrected absorbance (Abs_corr_) was calculated as:



Relative protein levels (RPL) were calculated relative to the mean protein level in all the sampled eggstrings using the following formula:



Where Abs_corr*sample _is the Abs_corr _for a protein sample at a given dilution and Abscorr_*dilution mean*dilution _is the mean Abs_corr _for all samples at that dilution. The c.i. was calculated from four parallel RPL values for each of the four dilutions. Pilot studies using the above design and the described peptide antiserum as well as five additional peptide antisera at various dilutions were conducted in advance to enable selection of the best serum and appropriate dilutions.

### Identification of LsTryp10 protein localization

Developing embryos from eggstrings 5 days post fertilization were dissected and incubated in 4% paraformaldehyde for 2 hours. Embryos were washed in PBS-AT (PBS, 1% albumin, 0.5% Triton-X) followed by blocking for 2 hours in 3% albumin, PBS, 0.5% Triton-X. The embryos were subsequently incubated with the antiserum used for ELISA (as described above) diluted 1:100 for 20 hours and washed in PBS-AT for 2 hours followed by incubation with Alexa Flour 594 conjugated goat-anti rabbit antibody (Invitrogen, Carlsbad, California, USA.) diluted 1:200 over night. Samples were mounted using ProLong Gold with DAPI (Invitrogen, Carlsbad, California, USA.) Paraffin sections (3 μm) were processed according to standard protocols. Slides were blocked with 3% albumin and incubated for 16 hours with the above mentioned antiserum. Alexa Flour 594 conjugated goat-anti rabbit antibody (Invitrogen, Carlsbad, California, USA.) diluted 1:200 was used as a secondary antibody. Samples were mounted using ProLong Gold(Invitrogen, Carlsbad, California, USA.). Confocal imaging was performed with a Leica TCS SP2 AOBS microscope (Leica Microsystems, Wetzlar, Germany) at the Molecular Imaging Center (FUGE, Norwegian Research Council, University of Bergen). Parallel negative controls were performed without primary antibody. At similar confocal settings no signals were detected from negative controls (except for signal from DAPI where present).

### Embryonic LysoTracker assays

Developing embryos were obtained as described in the previous section. The dissected eggs were immediately incubated in filtered sea water with 50 nM LysoTracker (Invitrogen, Carlsbad, California, USA.) for 30 minutes. Embryos were then briefly washed in seawater and visualized as described above.

## Authors' contributions

RSM and FN concieved the study, performed the sequence analyses and drafted the manuscript. RSM also performed quantitative real time PCR, *in situ *hybridization and protein quantification. PF helped coordinate the study and draft the manuscript. SD performed the immunohistochemical studies and helped to draft the manuscript. BOK sequenced cDNA and genomic DNA and helped draft the manuscript. IS helped quantifying protein levels. All authors read and approved the final manuscript.
